# American Diplomas in England

**Published:** 1893-08

**Authors:** 


### Editorial.

### AMERICAN DIPLOMAS IN ENGLAND.

In our last number we alluded briefly to the fact that the General Medical Council of Great Britain had decided to refuse registration to all American graduates. Under the proper heading there is given the memorial from the British Dental Association, an abstract from the report of the Educational Committee of the Medical Council, and the official order.

The course adopted is certainly the only consistent one for the Medical Council to take. In a former number this policy was advocated as the one that would probably prove satisfactory to the schools of this country and avoid much friction in English dental circles.

The reason given for the original acceptance of two colleges to the exclusion of others in this country is doubtless the true one, but was certainly formed on incomplete information at the time it was originated. The adoption of the original law of the Council, based as it was on an unjust discrimination, naturally led to unpleasant feeling on this side of the ocean, and hence it is eminently proper that it should be repealed and all the schools in the United States placed on one common level as far as practice in Great Britain is concerned.

This question of the recognition of foreign diplomas is a very broad one, and concerns all civilized communities. The criticism frequently made against foreign countries for their unwillingness to recognize diplomas of those outside their jurisdiction applies with equal force to some of the States of the United States where a similar condition of affairs exists.

While we have no sympathy with States that enact laws discriminating against others in the same confederacy, we can recognize that it is the duty of all well-regulated governments to protect as well as punish their own people, and inasmuch as this power is limited by their own boundaries, it becomes their duty to act within that limit with force and consistency. Upon this basis Germany refuses to recognize any foreign degree, and, we think, very properly.

To those affected by this law of exclusion, it seems unaccountable that intelligence, skill, and culture should not be recognized the world over. While it is true that there are only shades of difference between the training, professionally, of any of the civilized nations, and all thus trained can meet on common ground everywhere, as in our international conventions, it remains a fact that there are marked differences in the modes taken of reaching this standard of culture.

The foreign student, whether on the Continent, in Great Britain, or in the colonies connected therewith, must go through a regular classical training to reach any of the professions. In the United States greater stress is laid upon practical developments and less upon that deemed so important in England and elsewhere.

Which of these two positions is the correct one it is difficult to determine. There is evidently room for a variety of opinion and facts sufficient to lead to dogmatic utterances from whichever side the view be taken.

If results are alone to be considered, it may be questioned whether any country can take precedence. All can claim success in certain directions and have equally failed in others. This much must be conceded. It is clearly evident that the time spent in gymnasium and college is not conducive to a higher education in the practical branches necessary for skill in dentistry, and it is equally apparent that the training in what is termed the ordinary branches of education does not fit a man for the scientific culture so necessary in professional work. There will be found exceptions on both sides, the man of higher education, naturally inclined to mechanics, will drop into the proper groove and eventually become not only a good operator and mechanician, but a cultured scientist as well, and the poorly-educated man, through his higher aspirations and unwearied toil, will reach the same position. These exceptions are not, however, to be relied upon by governments in regulating the work to be performed.

All extremes have proved unsatisfactory, and we are forced to the conclusion that each profession or calling should establish a standard of preliminary training suited to its needs. Something similar to this is recognized in Germany in forming the three classes of schools,—the Gymnasium to train for the universities, the Real Schule for the polytechnic college, the Bürger Schule for the tradesmen and workers generally.

It would seem that a further subdivision should be adopted in the training for different callings. The elective system of some of the universities aims to meet this demand, and it is very satisfactory so far as it goes.

It is very evident that the dentist of the future will not need a Gymnasium or an Oxford or Cambridge training to fit him for the practice of his profession, nor is the work of the common schools adapted to qualify for the curriculum now required. The first overeducates in certain directions, and the other dwarfs the mentality and narrows the views of life.

As the older countries are fixed in their methods, and the United States is undergoing a transition period, an era of experimentation, as it were, it is impossible that there can be harmony in methods. While this condition exists it is proper that each country should stand upon its own ideas and exclude all who cannot meet the demands made.

Entertaining this view, we are decidedly of the opinion that the United States should meet this action on the part of foreign governments by a similar course of procedure. We have not been afflicted with the conservatism of the old civilizations, and have permitted all kinds of people and have welcomed all professions, and have opened freely the doors of our colleges and higher institutions of learning. On broad, generous principles this is right, but there comes a time when the reciprocity of political science may be safely adopted upon professional lines of work, and that time seems to be nearly at hand. We could say to the people of the Old World, "When you will take down your bars we will lower ours, but until that is done we will not recognize your diplomas nor receive your scientific men to the exclusion or injury of our own people."

We think this important question should be met, for our specialty, in the Association of Dental Faculties and the National Board of Dental Examiners, and should be viewed in no narrow, sectional spirit. The world must eventually come to a comprehension of the truth that the spirit of Chinese exclusiveness will not answer for the enlarged sphere which the active thought of the future must take, and lines of education based on egotistic assumption of superiority will not be the best foundation upon which to build professions.

